# Crystal Growth of Ca_3_Nb(Ga_1−*x*_Al*_x_*)_3_Si_2_O_14_ Piezoelectric Single Crystals with Various Al Concentrations

**DOI:** 10.3390/ma8095264

**Published:** 2015-08-26

**Authors:** Yuui Yokota, Tetsuo Kudo, Yuji Ohashi, Andrey Medvedev, Shunsuke Kurosawa, Kei Kamada, Akira Yoshikawa

**Affiliations:** 1 New Industry Creation Hatchery Center (NICHe), Tohoku University, 6-6-10, Aoba, Aramaki, Aoba-ku, Miyagi, Sendai 980-8579, Japan; E-Mails: kurosawa@imr.tohoku.ac.jp (S.K.); kamada@imr.tohoku.ac.jp (K.K.); yoshikawa@imr.tohoku.ac.jp (A.Y.); 2 Institute for Materials Research, Tohoku University, 2-1-1, Katahira, Aoba-ku, Miyagi, Sendai 980-8577, Japan; E-Mails: t_kudo@imr.tohoku.ac.jp (T.K.); ohashi@imr.tohoku.ac.jp (Y.O.); medvedev@imr.tohoku.ac.jp (A.M.); 3 C&A Corporation 6-6-40, Aoba, Aramaki, Aoba-ku, Miyagi, Sendai 980-8579, Japan

**Keywords:** langasite, piezoelectric crystal, single crystal, micro-pulling-down method

## Abstract

Ca_3_Nb(Ga_1−*x*_Al*_x_*)_3_Si_2_O_14_ (CNGAS) single crystals with various Al concentrations were grown by a micro-pulling-down (µ-PD) method and their crystal structures, chemical compositions, crystallinities were investigated. CNGAS crystals with *x* = 0.2, 0.4 and 0.6 indicated a single phase of langasite-type structure without any secondary phases. In contrast, the crystals with *x* = 0.8 and 1 included some secondary phases in addition to the langasite-type phase. Lattice parameters, *a*- and *c*-axes lengths, of the langasite-type phase systematically decreased with an increase of Al concentration. The results of chemical composition analysis revealed that the actual Al concentrations in as-grown crystals were almost consistent with the nominal compositions. In addition, there was no large segregation of each cation along the growth direction.

## 1. Introduction

Ca_3_NbGa_3_Si_2_O_14_ (CNGS) single crystal with an ordered langasite-type structure has been investigated as a piezoelectric material for some applications [1,2,3,4,5,6,7,8,9,10]. One of the expected applications is a combustion sensor in an engine of car or ship due to the relatively high piezoelectric constant and electromechanical coupling factor at high temperature. In addition, the electrical resistivity at high temperature of CNGS single crystal is higher than that of present crystals with a disordered langasite-type structure as represented by La_3_Ta_0.5_Ga_5.5_O_14_ (LTG), La_3_Nb_0.5_Ga_5.5_O_14_ (LNG), and La_3_Ga_5_SiO_14_ (LGS) single crystals [5]. In recent years, the CNGS crystals have also been investigated as an oscillator for the growing demand of the oscillator with small-size and low power consumption in low frequency range [6]. The temperature coefficient of frequency (TCF) on CNGS crystal is comparable to that of quartz along with the lower crystal impedance than quartz. Therefore, the CNGS crystal is expected to achieve the small-size oscillator with low power consumption in low frequency range. In our previous report, piezoelectric constant of an X-cut specimen was reported for the CNGS single crystal [7]. The piezoelectric constant *d*_11_ of the X-cut CNGS specimen was 3.98 pC/N and it was almost twice larger than that of quartz. The *d*_11_ was constant from room temperature to more than 500 °C and it is expected to be used at such high temperature that the quartz could not be applied. In addition, the TCF of a −24.5° Y-cut CNGS crystal in a temperature range from −10 °C to 70 °C were 60 ppm and it was smaller than that of the LGS crystal [6]. The great TCF of the CNGS crystal comparable to the AT-cut α-quartz is suitable for some applications as represented by the oscillator and the resonator.

We have developed shape-controlled langasite-type piezoelectric crystals by a micro-pulling-down (µ-PD) method [7,8,9,10,11]. The µ-PD method can control a shape of grown crystal using a special-shaped crucible [12] while conventional methods such as Czochralski (Cz), Bridgman-Stockbarger (BS), Floating Zone (FZ) and Flux methods cannot grow shape-controlled crystals. By the µ-PD method with special-shaped Pt crucibles, columnar, plate and tube-shaped langasite-type crystals have been grown and the shape-controlled langasite-type crystals were comparable in crystal quality and piezoelectric properties to CNGS crystals which were grown by Cz method. The near-net-shape growth of single crystal enables us to make piezoelectric elements with a configuration of final device element for each application and it can decrease costs of forming processes such as cutting and polishing after crystal growth. In addition, the µ-PD method can grow a single crystal with approximately 10 times faster growth rate than the conventional methods and it is also suitable for the material research of single crystal.

Effects of Al substitution to Ga site in langasite-type crystals with the disordered structure have been investigated [13,14,15,16,17]. In the case of LTG, LNG and LGS crystals with the disordered structure, a portion of Ga site can be substituted by Al ion and some piezoelectric properties such as electrical resistivity and piezoelectric constants were improved by the Al doping. In contrast, as for the CNGS crystal, there are reports only about full substitution for Ga sites by Al ions [5] and there is no detailed description about the Al concentration dependence of crystal growth, phase, crystal structure, chemical composition and physical properties for Al doped CNGS crystals. Especially in the case of the application for oscillator, the stability of TCF is one of the most important factors and the stability of TCF relates to more than one physical constant. Therefore, there is a possibility that Al doped CNGS crystal with a specific Al concentration indicates most suitable TCF for the application of oscillator.

In this study, we grew Al doped CNGS crystals with various Al concentrations by the µ-PD method and effects of Al doping on crystal growth, phase, crystal structure, chemical composition and electrical resistivity were investigated to clarify the effects of Al substitution on crystal growth, phase formation, segregation of cations and electrical resistivity at high temperature.

## 2. Experimental

Al doped CNGS (CNGAS) crystals with various Al concentrations were grown by the µ-PD method. Starting materials, CaCO_3_, Nb_2_O_5_, Ga_2_O_3_, SiO_2_ and Al_2_O_3_ powers (>4 N purity) were mixed as nominal compositions of Ca_3_Nb(Ga_1−*x*_Al*_x_*)_3_Si_2_O_14_ with *x* = 0.2, 0.4, 0.6, 0.8 and 1. The mixed powders were sintered at 1200 °C for 12 h in air several times and the sintered powders were set in a Pt-Rh crucible. The crucible has a ϕ 5 mm nozzle with a ϕ 0.5 mm capillary at the bottom. The crucible was placed in the center of a high-frequency (HF) induction coil and aluminum insulators surrounded the crucible. The crucible was heated in air up to the melting point of CNGAS by the HF coil and the mixed powder in the crucible was melted. Crystal growth was performed by pulling-down the melt on the bottom of the nozzle and LTG crystal was used as a seed crystal. Liquid-solid interface during crystal growth was observed by Charge Coupled Device (CCD) camera through holes of an after heater and insulators.

Phase and lattice parameters of as-grown crystals were identified by the powder X-ray diffraction (XRD) measurement using parts of as-grown CNGAS crystals. Crystals were sufficiently grounded by an agate mortar. Lattice parameters, *a*- and *c*-axes lengths, of langasite-type phases were calculated from the powder XRD patterns using Si powder as an internal standard. Surfaces of the polished CNGAS crystals were observed by the Scanning Electron Microscope (SEM) using Back scattering electrons (BSE). Chemical composition of each phase in the BSE images was analyzed by the Electron Probe Micro-Analysis (EPMA) using standard samples. Electrical resistivity of polished crystal at high temperature was measured by two terminal measurements.

## 3. Results and Discussion

**Figure 1:**
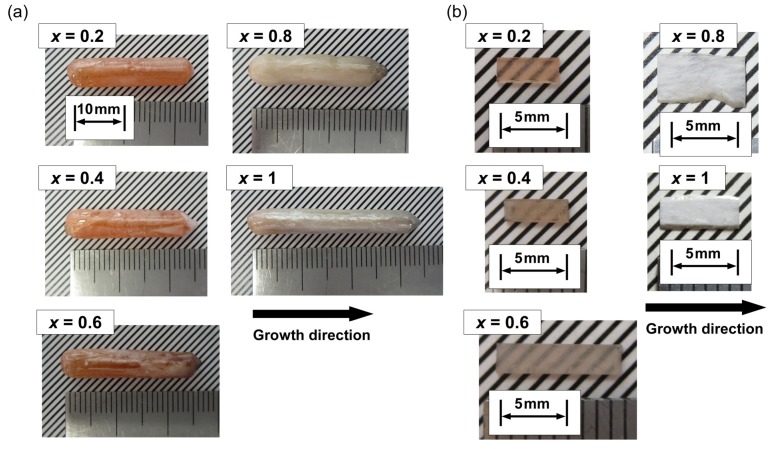
(**a**) As-grown Ca_3_Nb(Ga_1-*x*_Al*_x_*)_3_Si_2_O_14_ crystals grown by the micro-pulling-down (µ-PD) method; (**b**) Polished plate-like crystals cut from the as-grown Ca_3_Nb(Ga_1−*x*_Al*_x_*)_3_Si_2_O_14_ (CNGAS) crystals.

Liquid-solid interface was flat and stable just below the nozzle of crucible during crystal growth. Thickness of the melt between the liquid-solid interface and the bottom of the nozzle was approximately 100~200 µm. In the result, CNGAS crystals with a diameter of 5 mm and a length of several centimeters were obtained as shown in Figure 1a. The as-grown CNGAS crystals with *x* = 0.2, 0.4 and 0.6 indicated orange-color. The orange-color of the grown crystals is considered to be due to the oxygen defects. In our previous report about the effects of growth atmosphere for CNGS crystal, we reported the orange-color of grown crystal originated from the absorption around 300 nm in the transmittance spectrum [18]. On the other hand, as-grown crystals with *x* = 0.8 and 1 included milky parts in the crystals. The polished CNGAS crystals with *x* = 0.2, 0.4 and 0.6 were transparent while the crystals with *x* = 0.8 and 1 were opacity.

**Figure 2:**
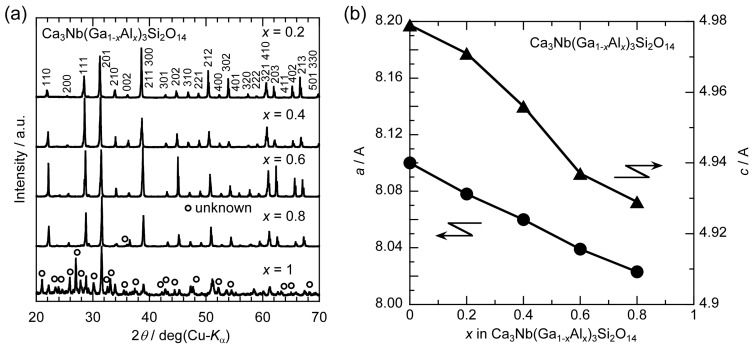
(**a**) Powder XRD patterns of the Ca_3_Nb(Ga_1−*x*_Al*_x_*)_3_Si_2_O_14_ crystals; (**b**) Lattice parameters, *a*- and *c*-axes lengths, of langasite-type phases for the CNGAS crystals calculated from the powder XRD patterns.

Powder XRD patterns of the as-grown CNGAS crystals were measured in order to investigate their phases and lattice parameters as it is illustrated in Figure 2a. All diffraction peaks in the XRD patterns of the CNGAS crystals with *x* = 0.2, 0.4 and 0.6 were indexed by the langasite-type structure and any secondary phases were not observed. In contrast, the XRD patterns of the CNGAS crystals with *x* = 0.8 and 1 included some secondary phases in addition to the langasite-type phase. Lattice parameters, *a*- and *c*-axes lengths, of langasite-type phases in the *x* range of 0.2~0.8 could be calculated from the XRD patterns (Figure 2b) *a*- and *c*-axes lengths systematically decreased with an increase of Al concentration and the result suggests that Ga sites in CNGAS crystals were substituted by Al ions with smaller ionic radius than Ga ion.

**Figure 3:**
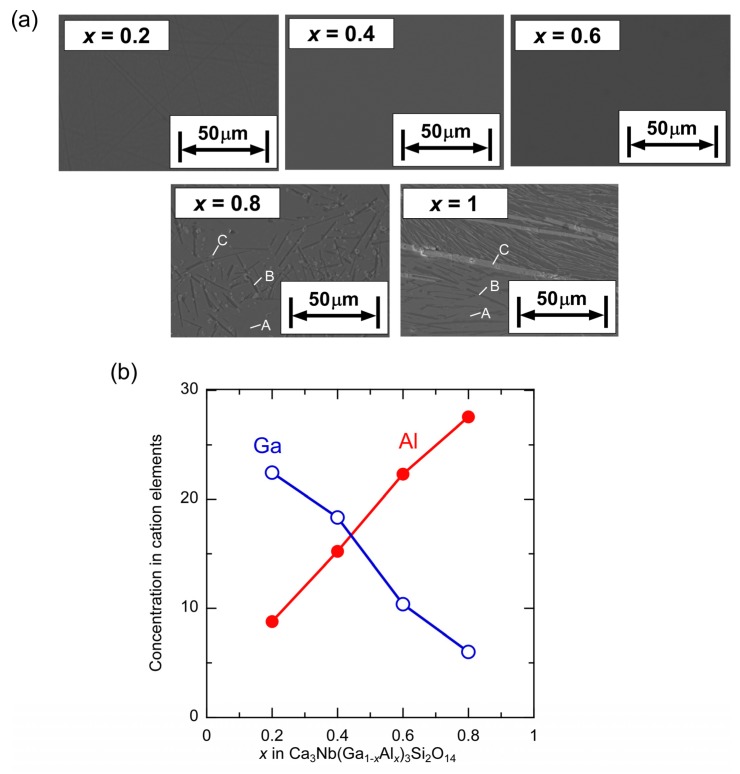
(**a**) Back-Scattering electron images of cross sectional planes for polished Ca_3_Nb(Ga_1__−*x*_Al*_x_*)_3_Si_2_O_14_ crystals. A, B and C were different phases; (**b**) Actual Ga and Al concentrations in cation elements of the langasite-type phase in the Ca_3_Nb(Ga_1−*x*_Al*_x_*)_3_Si_2_O_14_ crystals with *x* = 0.2, 0.4, 0.6 and 0.8.

Cross-sectional surfaces of the polished CNGAS crystals were observed by SEM with BSE (Figure 3a). For the CNGAS crystals with *x* = 0.2, 0.4 and 0.6, only one phase was observed in the BSE images and the result is consistent with the results of powder XRD patterns. In contrast, there were three phases in the BSE images of the CNGAS crystals with *x* = 0.8 and 1. Chemical compositions of their phases were analyzed by EPMA, and actual Al and Ga concentrations of the langasite-type phase in the CNGS crystals with *x* = 0.2, 0.4, 0.6 and 0.8 were illustrated in Figure 3b. Actual Al concentrations in the CNGAS phases were systematically increased and Ga concentrations were decreased with an increase of Al concentration in the nominal compositions. In the case of *x* = 0.8 and 1, gray areas (A in Figure 3) were langasite-type phase and the cation ratios were almost consistent with the nominal compositions. In contrast, black (B) and white (C) areas in BSE images of the crystals with *x* = 0.8 and 1 were identified by Ca-Al-Si-O and Ca-Nb-O systems, respectively. Increase of Al concentration in the CNGAS crystal generated secondary phases in the *x* range of 0.8~1 while even the crystal with *x* = 1 included the langasite-type phase, Ca_3_NbAl_3_Si_2_O_14_. The result suggests that the CNGAS was changed to incongruent phase from the congruent phase by the Al substitution and the change point is in the *x* range of 0.6~0.8.

**Figure 4:**
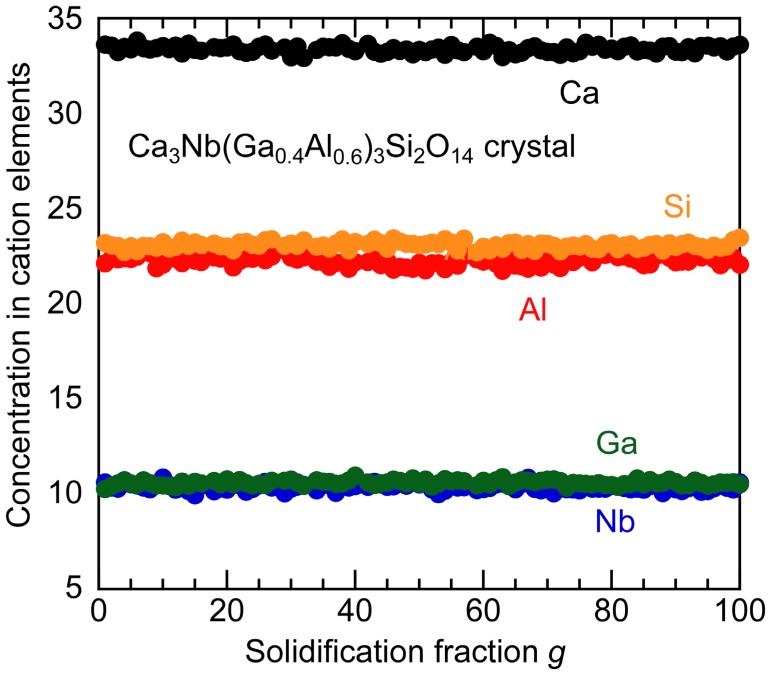
Chemical composition of cation elements along to growth direction for Ca_3_Nb(Ga_0.6_Al_0.4_)_3_Si_2_O_14_ crystal (*x* = 0.6) measured by Electron Probe Micro-Analysis (EPMA).

For the crystal with *x* = 0.6, chemical compositions of cation elements, Ca, Nb, Ga, Al, Si, along to growth direction were investigated. The segregation fraction dependence of each cation concentration is shown in Figure 4. All cations were almost constant with an increase of the solidification fraction. The effective solidification coefficient, *k*_eff_, of each cation was calculated by the standard segregation equation, *C* = C_0_*k*_eff_(1 − *g*)^*k*_eff_ − 1^ (*C*: cation concentration in the crystal, C_0_: cation concentration of the nominal composition, *g*: solidification fraction). In the result, all *k*_eff_’s of Ca, Nb, Ga, Al and Si were 1.00 and there was no segregation along to the growth direction in the grown crystal. However, actual Al concentration on Ga site in the crystal was approximately 68% and it was slightly larger than nominal composition. The essential reason for the increase of Al concentration in the grown crystal is not clear; however, there is a possibility that Ga evaporation during crystal growth increased the actual Al concentration in the crystal. The average chemical composition of grown CNGAS crystal was Ca_3.01_Nb_0.95_(Ga_0.32_Al_0.68_)_2.94_Si_2.08_O_14±δ_. In the result, Nb and (Ga,Al) concentrations were smaller than the nominal composition while Ca and Si concentration were larger than the nominal composition. The result suggests that there are some anti-site defects among caion sites in the crystal.

**Figure 5:**
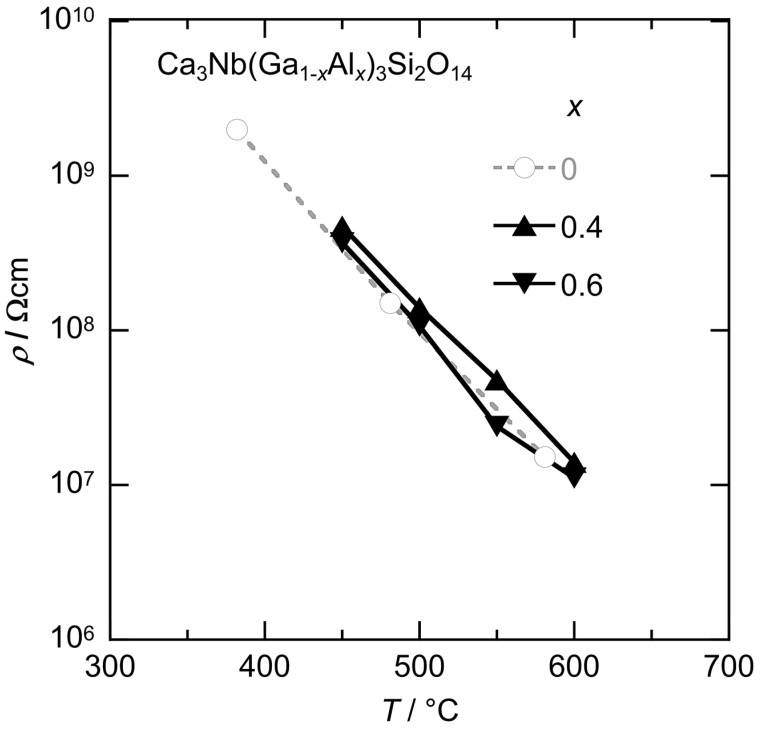
Temperature dependence of electrical resistivities for the Ca_3_Nb(Ga_1−*x*_Al*_x_*)_3_Si_2_O_14_ crystals with *x* = 0.4, 0.6 and undoped CNGS crystals.

Temperature dependences of electrical resistivities for the polished CNGAS crystals with *x* = 0.4 and 0.6 were described in Figure 5 and the electrical resistivity of undoped crystal was added by way of comparison. Clear difference in the electrical resistivities between CNGS and CNGAS crystals was not observed while Al substitution increased the electrical resistivity in the case of langasite-type crystals with the disordered structure [15].

## 4. Conclusions

Ca_3_Nb(Ga_1−*x*_Al*_x_*)_3_Si_2_O_14_ crystals with various Al concentrations were grown by the µ-PD method and their phases, lattice parameters, chemical compositions and electrical resistivities were investigated. Columnar-shaped CNGAS crystals were obtained and polished CNGAS crystals with *x* = 0.2, 0.4 and 0.6 indicated high transparency. On the other hand, the polished crystals with *x* = 0.8 and 1.0 were milky without a transparent part. Powder XRD patterns of their grown crystals indicated that CNGAS crystals with *x* = 0.2, 0.4 and 0.6 were a single phase of langasite-type structure and the crystals with *x* = 0.8 and 1 included some secondary phases in addition to the langasite-type phase. Lattice parameters, *a*- and *c*-axes lengths, of langasite-type phase in the *x* range of 0.2~0.8 systematically decreased with an increase of Al concentration. Segregation dependence of actual cation compositions in the crystal with *x* = 0.6 indicated the each cation was constant regardless of solidification fraction. The differences of cations between actual chemical composition and nominal composition are considered to be due to the Ga evaporation during crystal growth and anti-site defects on the cation sites. The detailed structural analysis of grown crystal is needed to clarify the essential reasons for the differences. The clear effect of Al substitution on the electrical resistivities was not observed while Al substitution increased the electrical resistivity in the case of langasite-type crystals with the disordered structure. The result suggests that there is not full agreement on the effects of Al substitution between the langasite-type crystals with the ordered and disordered structures.
